# Study of diversity of mineral-forming bacteria in sabkha mats and sediments of mangrove forest in Qatar

**DOI:** 10.1016/j.btre.2023.e00811

**Published:** 2023-08-22

**Authors:** Toka Mahmoud Farhat, Zulfa Ali Al Disi, Mohammad Yousaf Ashfaq, Nabil Zouari

**Affiliations:** a Environmental Sciences Program, Department of Biological and Environmental Sciences, College of Arts and Sciences, Qatar University, P.O.B 2713, Doha Qatar; b Environmental Science Center, Qatar University, P.O. Box 2713, Doha, Qatar

**Keywords:** Sabkha, Mangroves, Biomineralization, MALDI-TOF MS, Biodiversity

## Abstract

The involvement of microorganisms in carbonate minerals and modern dolomite formation in evaporitic environments occupied with microbial mats (i.e., sabkha) and in mangrove forests is evidenced, while its potential diversity requires further elucidation. Microorganisms can create supersaturated microenvironments facilitating the formation of various carbonate minerals through specific metabolic pathways. This is particularly important in arid environments, where deposition and sedimentary structures can occur. This study investigated the biodiversity of halophilic, heterotrophic, and aerobic mineral-forming bacteria in mangrove forests and living and decaying mats of Qatari sabkha. The diversity study was performed at the protein level using MALDI-TOF mass spectrometry protein profiles combined with principal component analysis (PCA), which revealed a high diversity of isolated strains at the taxonomy and protein profile levels. The diversity of the minerals formed in pure cultures was evidenced by SEM/EDS and XRD analysis. Different types of carbonate minerals (calcium carbonate, magnesium carbonates, and high-magnesium calcites) were formed in pure cultures of the studied strains, which might explain their occurrence in the bulk composition of the sediments from where the strains were isolated. These results illuminate the diversity of biological mineral-formation processes in the extreme environments of Qatari sabkhas and mangroves, explaining the high diversity of minerals in these environments.

## Highlights

• Microorganisms play a significant role in the formation of carbonate minerals in both evaporitic environments and mangrove forests.
• The study investigated the biodiversity mineral-forming bacteria in mangrove forests and Qatari sabkha through protein level analysis using MALDI-TOF MS com protein profiles combined with PCA.
• The diversity of the minerals formed in pure cultures was evidenced by SEM/EDS and XRD analysis.

## 1 Introduction

The coastline of Qatar consists of complex marine ecosystems including sabkhas and mangroves forests. Studies in both sabkha and mangrove habitats are important due to their sedimentary structure and biodiversity [1]. The supratidal sabkhas are unique with their characteristics as they are also defined as marine environments [2]. All these types of ecosystems exist also in the coastal regions of other areas like the Mediterranean area [3]. However, along the Arabian Gulf coast, sabkhas are mostly evaporitic areas, representing specific sites of diversity due to the harsh weather conditions [4]. Indeed, the average temperature of the sea surface in the Arabian Gulf Sea fluctuates around 20 – 34 °C [5], while the temperature above the surface of the sedimentary structures may reach 60 °C in the summer. Consequently, the salinity can rise to 30% [6]. The high salinity can contribute to the formation of minerals in sabkhas as well as to the role of mineral-forming bacteria evidenced in these evaporitic areas [7].

Various environments can be ideal for the existence of microbial mats [8]. The coastal intertidal sediments and sabkhas are considered one of those environments, contributing significantly to improving the sedimentary deposits. The microbial mats in sabkhas serve as an important natural laboratory for studying the role of the mineral-forming microorganisms in the evaporitic mineralization [9], [10], [11]. The various microorganisms living in sabkha mats can perform highly diversified metabolic activities as evidenced inside these microbial mats [12]. The microbial mats found in sabkha regions are not only distinctives ecosystems, but also dynamic ones as they constitute enormous biodiversity of living microorganisms, which can shape the assembly of the sediments within the environment [13]. On the other hand, sabkhas are considered vulnerable to climate change, due to the lack of freshwater supplies and rainfall as well as increase of human population [14]. Although the fluctuations of the harsh conditions that characterize the Arabian Gulf areas, it was demonstrated that the marine habitats including mangroves forests, sabkhas and seagrasses still can serve as CO_2_ sinks [15], and in turn, can affect the “blue carbon,” which refers to carbon stored in marine environments [16].

Mangroves are a group of trees and shrubs that grow in coastal saline or brackish water habitats in tropical and subtropical regions [17]. They have the ability to endure a variety of challenging conditions, including minimal rainfall, intense sunlight, significant temperature fluctuations, and extreme salinity [18]. Symbiosis between microorganisms, developed in their rhizosphere, might be one of the crucial factors of their existence [19]. In addition, sediments under mangrove forests are shown as great reactors for the formation of minerals [20]. Their presence in tropical and subtropical areas provides all the factors necessary for the formation of minerals [21]. The later work showed that the activities of mineral formation and biotransformation were performed by bacteria isolated from sediments of mangroves forests. The mangrove forest of Simaisma in Qatar is one of the mangrove-rich zones in the coastline of Qatar. The specific Qatari conditions ensure a strong adaptation within the microbial communities, leading to new biological activities, of interest [22].

MALDI-TOF MS (Matrix-Assisted Laser Desorption/Ionization Time-Of-Flight Mass Spectrometry) is a powerful analytical technique that enables the rapid and accurate identification of proteins, peptides, and other biomolecules based on their mass-to-charge ratio (*m/z*) [23]. The technique has a wide range of applications in fields such as proteomics, drug discovery, and clinical diagnostics [24], [25], [26]. The MALDI-TOF MS technique has several advantages, for example, it is relatively fast and easy to use, requires minimal sample preparation, and can analyze a wide range of sample types, including proteins, peptides, lipids, and carbohydrates [27]. Recently, it was shown efficient in the identification, differentiation and categorization of mixtures of bacteria living in the environment as consortia even at the species levels [7,28]. It becomes a useful approach for the study of functional biodiversity in environmental studies.

Here, mineral-forming bacteria were isolated form microbial mats of Qatari sabkha and sediments of mangrove forests. By using MALDI-TOF MS to study their biodiversity, a new collection of mineral-forming bacteria from both environments could be examined. This would provide insight into the diversity of these bacteria at the genus and species levels, as well as their metabolic proteins which express their adaptive diversity. The adaptations of these bacteria are linked to their spontaneous biodiversity. By combining bacterial identification and protein profiling, with a focus on protein markers discovered during mineral formation, these bacteria can be classified as biomineral-forming bacteria. This classification represents important evidence of biodiversity related to the adaptation of mineral-forming bacteria and demonstrates that Qatari sabkhas and mangrove sediments form a continuous, dynamic bacterial system for capturing CO_2_.

## 2 Material and methods

### 2.1 Sampling sites

**Fig. 1:**
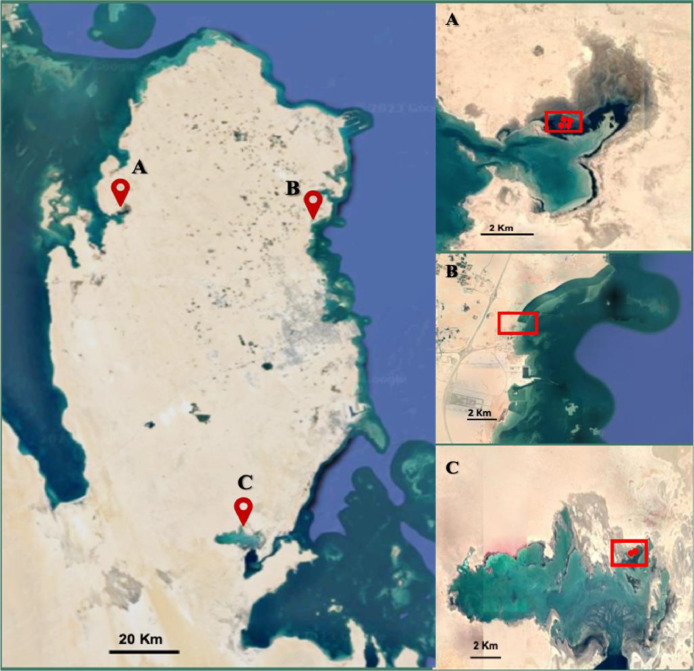
Qatar's map with locations of sampling: A) Dohat Faishakh sabkha, B) Simaisma mangroves and C) Khor Al-Adaid sabkha.

The sampling locations in two sabkhas were chosen based on prior research that confirmed the existence of various types of precipitates and forecasted the likelihood of dolomite formation [10,11]. Khor Al-Adaid sabkha is a settlement existing in southeast region of Qatar in Alwakrah municipality (GPS coordinates 24°38′45.870″N 51°19′35.760″E). Dohat Faishakh sabkha is a bay located in Al-Rayyan municipality (GPS coordinates 25°38′8.17″N 50°57′36.48″E). Four samples of the microbial mats (DFC, DFD, DFL and DFM) were collected aseptically from intertidal zone of Dohat Faishakh sabkha (10 m apart) and one microbial mat (KA1) was collected from Khor Al-Adaid sabkha. Another sampling location was in Simaisma mangrove forests existing in Al Daayen municipality, eastern region of Qatar, (GPS coordinates 25°34′41.1″N 51°29′19.1″E). Sediment samples were collected in a sterile 50 mL Falcon tube. The samples were collected from a depth of 20 cm under two mangrove trees (MC1 and MC2) and at 1 m far from each mangrove tree (MC1F and MC2F). Upon collection, all samples were temporarily kept in an icebox at 4 °C. The samples were transferred to the laboratory and then preserved at −20 °C for further analysis. The three sampling locations are illustrated in Fig. 1.

### 2.2 Culture media

The liquid MD1 medium is composed of (g/L): 10 yeast extract, 5 peptone, 1 glucose, 12 magnesium acetate, 1.5 calcium acetate and 35 sodium chloride. Solid MD1 medium was prepared by adding 15 g/L agar. The pH was adjusted to 7.0 before sterilization at 121°C for 20 min. Luria-Bertani (LB) medium is composed of (g/L): 10 tryptone, 5 yeast extract, 10 sodium chloride added to 1 L of distilled water. Solid LB contains 15 g of agar in addition to the former for 1 L. LB media were autoclaved at 121 °C for 20 min.

### 2.3 Isolation and preservation of the mineral-forming bacterial strains

Mineral-forming bacterial strains were isolated from all the sabkhas and mangrove sediments by the process of enrichment cultures. The medium MD1 was used for the growth of this category of bacteria [10]. One g of sample was suspended in 20 mL MD1 and incubated for 72 h in a rotary shaker set at 30 °C and 150 rpm. Four successive enrichment cultures were then proceeded under the same conditions, each inoculated with 2 mL from the former culture. Serial dilutions were plated from the last culture on solid MD1 and the dilutions allowing for selection of separate colonies. Distinct colonies were transferred to a new MD1 plates using the streaked plate method. Then, five successive sub-cultures of a separate colony from each, allowed purification of each strain. The isolated strains were given codes and preserved at −80 °C in 60% glycerol in LB medium. Before being used to inoculate pure cultures, each strain was plated in LB medium and separate colonies were used.

### 2.4 Identification of the isolated strains by MALDI-TOF MS

For identifying and generating the protein profile of each of the isolated strains by MALDI-TOF MS, they were cultured on LB solid medium and incubated overnight at 30 °C. MALDI-TOF MS was used as described by Abdel Samad et al. (2020). It generates a specific mass spectrum related to the proteins of each strain, then compared with the database entries. Proteins having a *m/z* between 2000 and 20,000 are utilized to identify the bacterial strain based on individual mass peaks matching to specific ribosomal proteins, available in a database provided in the software. The data are presented as log (scores) by the Biotyper software which generates them by default. For each strain, a log scale ranging from 0.000 to 3.000 was attained. The identification is at the extremely probable species level with high confidence if the score falls between 2.300 and 3.000. The scores between 2.000 and 2.299 offer highly accurate genus-level identification and probable correct species-level identification. Probabilistic genus level identification is provided by scores between 1.700 and 1.999. Analysis and protein profiles were performed with the MALDI Biotarget-48 sample spots using MALDI Biotyper Real time classification software. The manual process of protein profiles was performed using the Flex Analysis Software which is necessary for smoothing and subtraction of baseline from the profile.

### 2.5 Differentiation and diversity study of the isolated strains by MALDI-TOF MS and PCA

Principle component analysis (PCA) was used in this study in order to reduce the effect of the dimensionality of the data set maintaining thus the original information. PCA is a statistical technique used to transform a dataset into a lower-dimensional space by identifying the principal components. Each principal component is a linear combination of the original variables and represents a specific pattern or direction of variability in the data. The components are ordered in terms of the amount of variance they explain, with the first component explaining the most variance, the second component explaining the second most, and so on. The PCA used the peaks obtained in each protein profile as generated by MALDI-TOF MS. In fact, the peaks which are not matching to specific ribosomal proteins, are of not identified proteins and peptides The results of PCA are interesting since the profiles are clustered into groups of similar variation characteristics allowing the visualization of the differences between the strains. Here, the data are represented in 3D coordinate system offering at least 50% of the total variance between the samples. This study was based on the standard operating procedure of the MALDI Biotyper instrument.

### 2.6 Identification of the isolated strains by ribotyping (16S rRNA)

DNA was extracted from cells that had grown overnight on LB plates at a temperature of 30 °C. In a sterile environment, a single colony of bacterial biomass was stripped from the surface of culture plates. The bacterial biomass was suspended in 500 μL distilled water, placed in water bath at 100 °C for 10 min, and then transferred immediately to −20°C for 10 min [29]. After centrifugation of 5 min at 10,000 rpm, the supernatants were transferred into new tubes. PCR mixtures with total volume of 25 μL were prepared by adding 3 μL of DNA for each sample, 2.5 μL of the two selected universal primers (RibS74sp 5′-AAGGAGGTGATCCAGCCGCA-3′ and RibS73sp 5′-AGAGTTTGATCCTGGCTCA-3′), Al Disi et al., 2017), 4.5 μL of nuclease-free water to 12.5 μL master mix containing: (1.5 μM MgCl_2_, 0.8 μM dNTP, 1.35 μM). The PCR reactions were performed using an Applied Biosystems instrument. The genomic DNA of the isolates were used as template for the PCR reactions. To initiate the PCR reactions, the first step involved an initial denaturation at 94 °C for three minutes. This was followed by 35 cycles consisting of denaturation at 94 °C for 45 s, annealing at 50 °C for 45 s, and elongation at 72 °C for 45 s. A final extension step was then performed at 72 °C for two min. Afterward, the DNA amplicons were purified with the Thermo Scientific GeneJET PCR Purification Kit. The Sanger sequencing was carried out in laboratories of Weill Cornell Medicine – Qatar. The obtained DNA sequences were compared to the most closely sequences available at NCBI Blast server.

### 2.7 Screening of the studied isolates for the mineral formation capabilities

The potential of the isolates to form minerals was evaluated using the MD1 and two modified solid media namely MD1P and MD1Y, as evidenced by Abdelsamad et al. (2022). The two modified media contain the same components of MD1 media but MD1P is made up without yeast extract and MD1Y without peptone. Each isolate was grown on the solid media and incubated at 30 °C for 3 weeks. The growth areas were monitored regularly with a light microscope to observe the formation of crystals.

### 2.8 Investigation of the minerals composition by X-Ray diffraction (XRD), screening electron microscopy and energy dispersive X-Ray (SEM/EDS) analysis

The sediment samples collected from sabkhas, and mangrove forest had been analyzed initially to ensure the existence of high magnesium carbonate as a bulk in these sites. One g from each sediment sample was manually grounded and homogenized with a mortar and pestle, mixed evenly, and left to dry at 37 °C overnight. The dried samples were analyzed by XRD and SEM/EDS.

The minerals formed in pure bacterial cultures were extracted from the solid media. The bacterial biomass was stripped from the surface of the solid medium by slightly scraping the top layer with a sterile scalpel. The mineral crystals were washed three times with 15 mL distilled water. This process does not alter the shape of the crystals, as confirmed by optical microscopy before and after recovery [10]. The overnight dried samples at 37 °C, were analyzed by SEM/EDS and XRD techniques.

The SEM analysis was conducted utilizing a Nova Nano Scanning Electron Microscopy equipped with a Bruker EDX Detector with a magnification of 200,000X and a resolution of 5 nm. The EDS was obtained in accordance with the “ASTM standard method E1508–12a”, using a spot size of 5 and an accelerating voltage of 20 kV with an error rate of 4%. The PANalytical- multipurpose Empyrean X-ray diffractometer was used to determine the bulk mineralogical composition of the retrieved minerals.

## 3 Results and discussions

### 3.1 Investigation of the occurrence of carbonate minerals in the mangrove sediments by XRD and SEM/EDS analysis

**Fig. 2:**
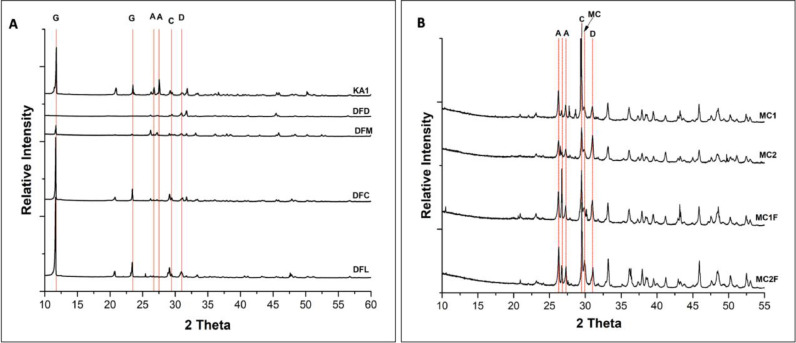
Representative XRD patterns of sediments sampled from A) Sabkha (DFC, DFD, DFM and DFL) and B) Simaisma mangroves (MC1, MC2, MC1F and MC2F) indicating the occurrence of different carbonate mineral phases. G: Gypsum, C: Calcite, A: Aragonite, D: Dolomite, MC: Magnesium calcite.

**Fig. 3:**
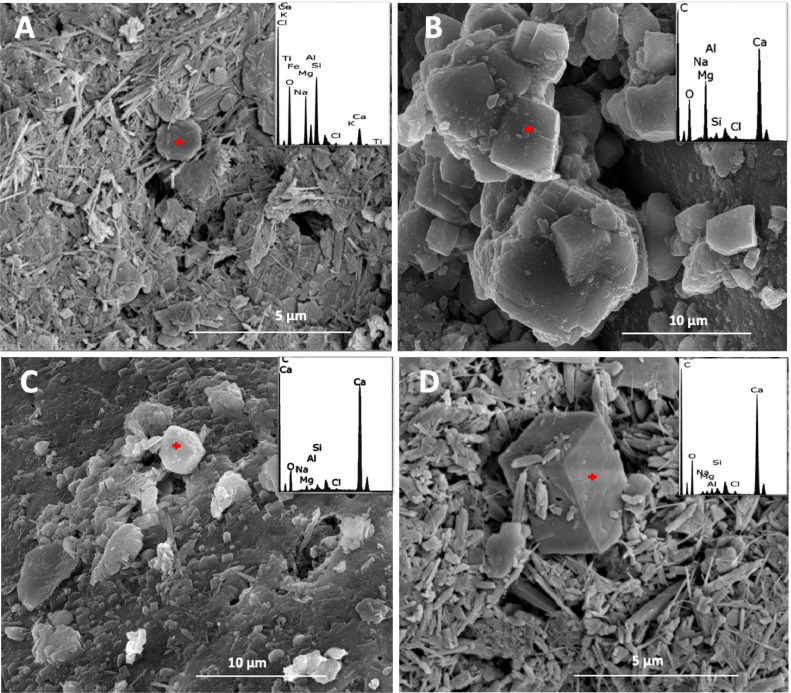
Representative SEM/EDX images of Khor Al-Adaid sabkha sediment (KA1), B) Dohat Faishakh sediment (DFM), C) Dohat Faishakh sediment (DFC), and D) and Simaisma mangrove sediment (MC23). EDX analysis, top right panels, indicate the elemental composition.

The occurrence of a variety of carbonate minerals, including calcium carbonates, magnesium carbonates and dolomite, was demonstrated earlier by XRD in the living mats and the decaying mats of both Sabkhas; Dohat Faishakh and Khor Al-Adaid sabkhas [10,11]. Here, these minerals were evidenced in sediments sampled from Sabkha and Simaisma mangroves. Fig. 2 shows the occurrence of different carbonate mineral phases in these samples. Indeed, the XRD analysis confirms the presence of a mixture of carbonate minerals including, aragonite, calcite, high-Mg calcite, and dolomite. The SEM/EDX was performed on all the samples from both sabkhas and mangrove sites, and the images of the bulk sediments are shown in Fig. 3. The results show the existence of carbonate minerals with different Mg^+2^: Ca^+2^ ratios in all the samples, including Khor Al-Adaid sabkha sediment KA1, Dohat Faishakh sediment DFM and Simaisma mangrove sediment MC23.

### 3.2 Identification of the isolated strains by MALDI-TOF MS and by ribotyping

Thirty-nine bacterial strains were isolated: five strains from living mats of Khor Al-Adaid, fourteen strains from decaying mats of Dohat Faishakh and twenty strains from Simaisma mangrove sediments. The identification of the thirty-nine isolated strains was performed using MALDI-TOF MS using the Bruker database entries [30]. Only nineteen strains were given a MALDI score higher than 1.7, which allowed their identification at the genus level and for certain ones, at the species level. The MALDI-TOF MS protein profiles of the others were not matching to the database entries. Indeed, the database of the used MALDI machine comprises the data related to bacteria, which are mostly searched in the health and environmental field, including those belonging to genera: *Shewanella, Staphylococcus, Vibrio, Micrococcus, Bacillus* and *Salinivibrio.* Out of the nineteen isolates identified by MALDI-TOF MS, five were identified as *Salinivibrio proteolyticus* and five as *Vibrio alginolyticus*, two of each *Bacillus subtilis, Bacillus lichenformis* and *Shewanella putrefaciens* and one of each *Bacillus seohaeanensis* and *Staphylococcus epidermidis* and *Micrococcus luteus.* Previously, strains belonging to these genera were isolated from different environments and identified by both techniques (MALDI-TOF MS using the same database and ribotyping) [7,24,27,28,[31], [32], [33], [34]]. The identification by ribotyping of the twenty strains which were not identified by MALDI-TOF MS was performed and the sequences were compared to the most closely sequences available at NCBI Blast server. Among these twenty isolated, five were *Virgibacillus chiguensis*, three *Virgibacillus dokdonensis*, two *Virgibacillus marismortui*, two *Virgibacillus sp,* two *Halomonas sp.*, one *Oceanimonas baumannii*, one *Bacillus swezeyi*, one *Pantoea agglomerans*, one *Halomonas Hydrothermalis*, one *Bacillus licheniformis*, one *Virgibacillus pantothenticus*, and one *Bacillus sp*. The two strains *Bacillus swezeyi*, DFD32 and *Bacillus licheniformis*, DFC22, were not identified by MALDI-TOF MS although this genus and subspecies are in the database of the MALDI software. This shows the high sensitivity of the MALD-TOF MS procedure in considering the specific proteins in the MALDI-scores determination. A score below 1.7 out 2.3 is not considered for the accurate identification. The use of MALDI-TOF in the differentiation of very close strains is efficient [31]. The two strains were identified by ribotyping. Table 1 indicates the isolates that were isolated from the three studied site and their identifications.

**Table 1:** List of the bacterial strains isolated from the decaying mats of Dohat Faishakh sabkha, the living mats from Khor Al-Adaid sabkha and the sediments of Simaisma mangroves and their identification (by MALDI-TOF MS and ribotyping).

No.	Source	Strain code	Identification by MALDI TOF	MALDI TOF score	Identification by ribotyping		Similarity%		GenBanck accession number			PCA No.				
1.	Mangrove Site MC1	MC104	Not identified	< 1.7	*Virgibacillus chiguensis*		97%		MN581194.1			64				
2.		MC113	*Shewanella putrefaciens*	1.92	*–*		–		–			9				
3.	Mangrove Site MC1F	MC1F32	Not identified	< 1.7	*Virgibacillus sp.*		97%		KT597078.1			30				
4.		MC1F33	Not identified	< 1.7	*Virgibacillus pantothenticus*		98%		KX957797.1			78				
5.	Mangrove Site MC2	MC211	Not identified	< 1.7	*Virgibacillus dokdonensis*		98%		MN581194.2			73				
6.		MC223	Not identified	< 1.7	*Virgibacillus chiguensis*		98%		MN581194.1			59				
7.		MC224	Not identified	< 1.7	*Halomonas sp.*		96%		AB305249.1			80				
8.		MC2251	Not identified	< 1.7	*Halomonas sp*.		98%		AB305249.1			5				
9.		MC23	Not identified	< 1.7	*Halomonas Hydrothermalis*		97%		AP022843.1			101				
10.		MC231	*Staphylococcus epidermidis*	2.2	*–*		–		–			47				
11.		MC233	Not identified	< 1.7	*Virgibacillus dokdonensis*		98%		MK622388.1			145				
12.		MC234	Not identified	< 1.7	*Virgibacillus sp.*		98%		KT597078.1			123				
13.	Mangrove Site MC2F	MC2F021	Not identified	< 1.7	*Oceanimonas baumannii*		82%		KX148511.1			79				
14.		MC2F022	Not identified	< 1.7	*Pantoea agglomerans*		74%		KJ529102.1			36				
15.		MC2F111	*Vibrio alginolyticus*		2.07	*–*			–		–			29		
16.		MC2F13	*Vibrio alginolyticus*		2.14	*–*			–		–			8		
17.		MC2F211	Not identified	< 1.7	*Virgibacillus dokdonensis*		98%		MK622388.1			94				
18.		MC2F212	Not identified	< 1.7	*Virgibacillus chiguensis*		98%		MN581194.1			74				
19.		MC2F31	*Shewanella putrefaciens*	1.96	*–*		–		–			27				
20.		MC2F34	*Micrococcus luteus*	2.35	*–*		–		–			57				
21.	Dohat Faishakh Sabkha	DFC02	Not identified	< 1.7	*Virgibacillus chiguensis*		98%		MN581194.1			76				
22.		DFC03	Not identified	< 1.7	*Virgibacillus marismortui*		98%		KU194376.1			58				
23.		DFC04	Not identified	< 1.7	*Virgibacillus marismortui*		97%		KU194376.2			4				
24.		DFC22	Not identified	< 1.7	*Bacillus licheniformis*		96%		HG799978.1			89				
25.		DFD111	*Bacillus subtilis*	1.77	*–*		–		–			142				
26.		DFD12	*Bacillus lichenformis*	1.87	*–*		–		–			87				
27.		DFD121	*Bacillus subtilis*	2.15	*–*		–		–			110				
28.		DFD32	Not identified	< 1.7	*Bacillus swezeyi*		98%		NR157608.1			107				
29.		DFL02	Not identified	< 1.7	*Virgibacillus chiguensis*		98%		MN581194.1			84				
30.		DFL03	Not identified	< 1.7	*Bacillus sp.*		86%		HG799978.1			41				
31.		DFL04	*Bacillus seohaeanensis*	2.07	*–*		–		–			67				
32.		DFL32	*Bacillus lichenformis*	1.74	*–*		–		–			111				
33.		DFM01	*Vibrio alginolyticus*		2.06	*–*			–		–			19		
34.		DFM03	*Vibrio alginolyticus*		2.09	*–*		–		–			16			
35.	Khor Al-Adaid Sabkha	KA102	*Vibrio alginolyticus*	1.99	*–*		–		–			25				
36.		KA103	*Salinivibrio proteolyticus*	2	*–*		–		–			3				
37.		KA104	*Salinivibrio proteolyticus*	2.14	*–*		–		-			37				
38.		KA105	*Salinivibrio proteolyticus*	1.87	*–*		–		–			20				
39.		KA106	*Salinivibrio proteolyticus*	2.2	*–*		–		–			22				

### 3.3 Investigation of the biodiversity of the bacterial strains isolated from Qatari sabkhas and mangrove sediments using PCA and dendrogram analysis

**Fig. 4:**
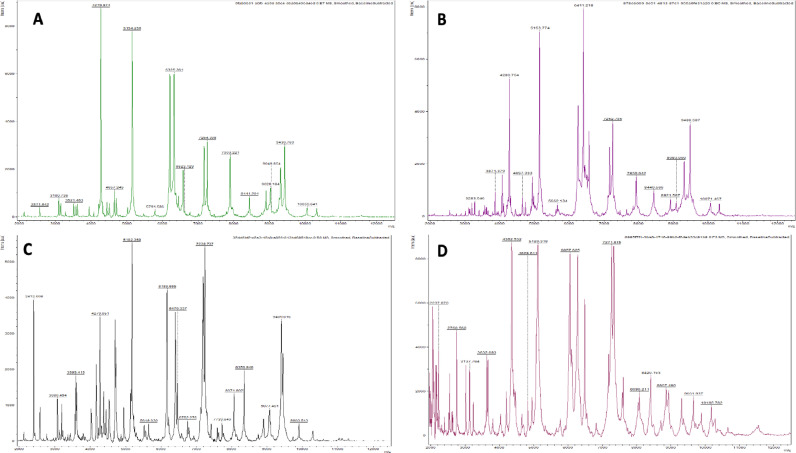
Samples of the protein profiles generated by MALDI-TOF MS for A) *Virgibacillus chiguensis* (MC223), B) *Vibrio alginolyticus* (MC2F13), C) *Virgibacillus marismortui* (DFC03) and D) *Salinivibrio proteolyticus* (KA106).

MALDI-TOF MS allowed obtaining the protein profile for each identified and not identified strain. Fig. 4 shows the protein profiles of several strains. The considered profiles are composed of proteins between 2000 and 20,000 *m/z*. Each peak from protein profiles is presenting one of the proteins that have been produced within the bacterial cell during growth.

**Fig. 5:**
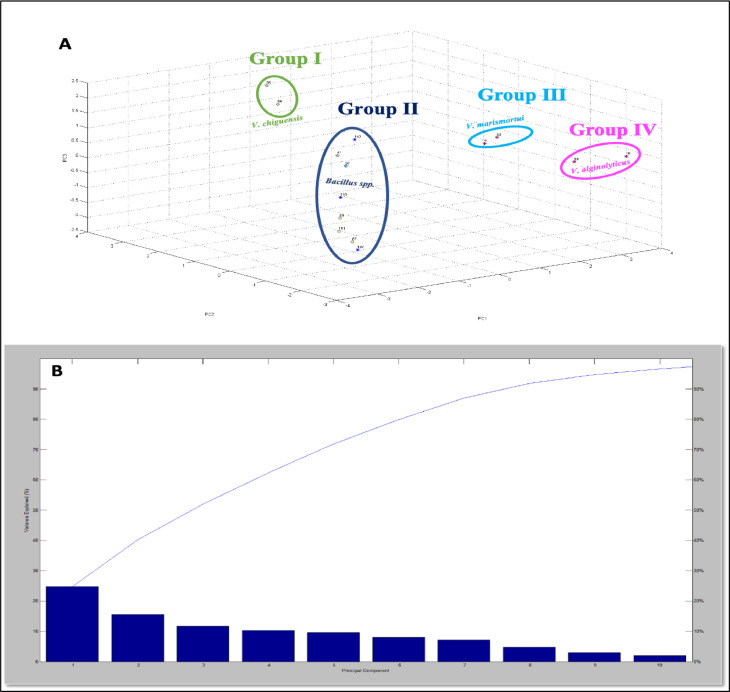
PCA classification of the isolates from Dohat Faishakh sabkha, A) PCA, B) percentage of variance explained by PCA.

Additional insights into the links between closely related isolates was performed by combining MALDI-TOF MS and PCA. PCA allowed the creation of linear combinations of variables to represent the objects being studied by reducing the dimensions of the objects. The percentage variance explained for the combined isolates of sabkhas, and mangroves revealed a total of 40% with contributions of PC1 (18%), PC2 (12.5%), and PC3 (9.5%). Consequently, the first three components were not considered to be sufficient to explain the variability of data. As the general rule, the PCA total variance should be at least 50% as recommended by Streiner (1994) [35]. The PCA was not performed for Khor Al-Adaid isolates because the number of isolated strains was only 5 strains enclosing only two species of bacteria. Therefore, the PCA clustering and the dendrogram were performed for data from Dohat Faishakh sabkha and Simaisma mangrove, individually. The PCA clustering for sabkha isolates demonstrated significant protein-level diversity among the analyzed strains. The variability of the principal components is shown in Fig. 5B, with PC1 (25%), PC2 (16%), and PC3 (13%) contributing to a combined 54% of the data variability. As for the principal component analysis, the differences at group level were demonstrated by the distance between clusters, whereas the variations in protein profiles were highlighted by the distance between the individual strains within clusters. Four clusters were established based on the PCA for Dohat Faishakh sabkha samples as shown in Fig. 5A. Cluster I covers *V. chiguensis*; cluster II contains *Bacillus spp.,* while Cluster III is formed of *V. marismortui* and cluster IV of *V. alginolyticus*.

**Fig. 6:**
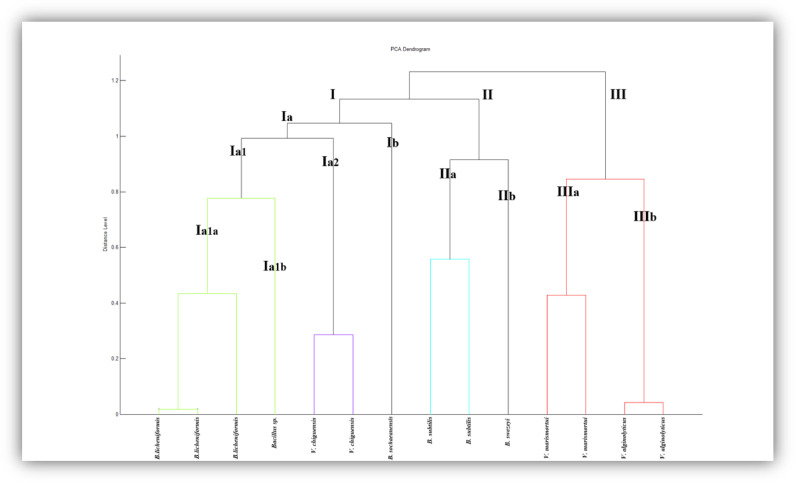
PCA dendrogram of all strains isolated from Dohat Faishakh sabkha.

To investigate the hierarchical relationship between the isolates, the PCA clusters and the dendrogram for Dohat Faishakh sabkha and mangroves strains were established. The dendrogram for Dohat Faishakh sabkha isolates, shown in Fig. 6, displays that the three main clusters I, II, and III are subdivided into several clades. Cluster I is made up of seven isolates, cluster II contains three isolates while cluster III consists of four strains. Cluster I consists of two main clades, Ia and Ib. The clade Ia is further divided into two sub-clades, Ia1 and Ia2. Within the sub-clade Ia1, there are three strains of *B. lichenformis* and Ia1b has one *B. subtilis* strain. Ia2 includes two *V. chiguensis* strains. The Ib clade covers *B. sochaeanensis* strain. Cluster II involves two clades IIa containing two *B. subtilis* strains and IIb having one *B. swezeyi* strain. Cluster III contains two clades: IIIa and IIIb. IIIa clade encloses two *V. marismortui* strains and IIIb involves two *V. alginolyticus* strains.

**Fig. 7:**
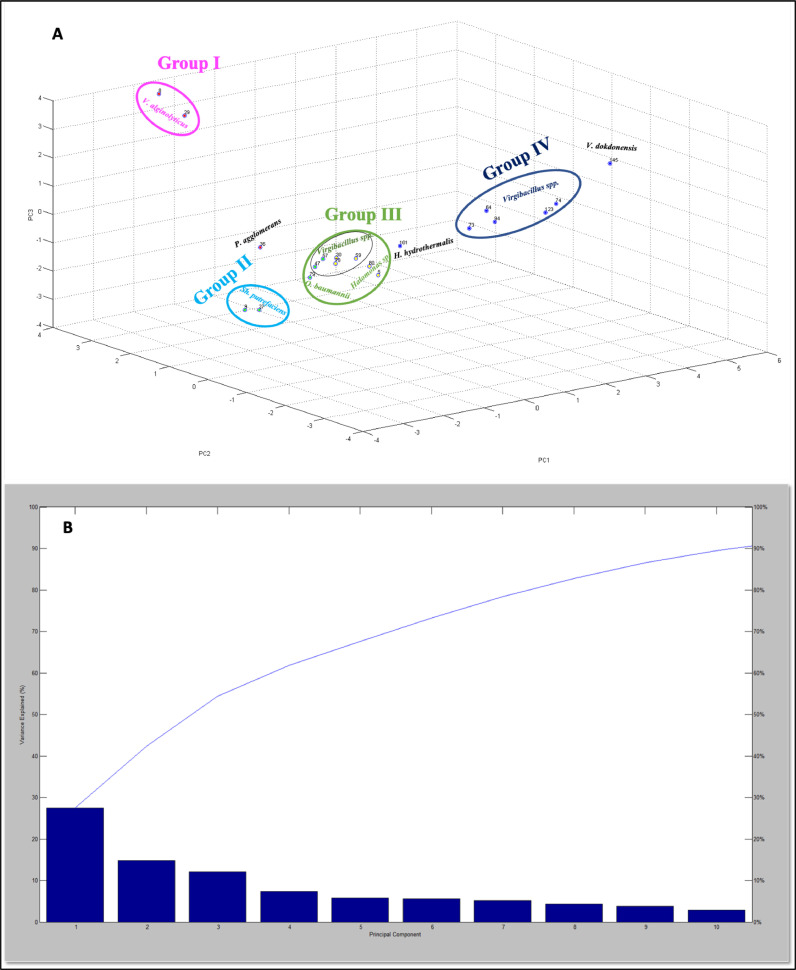
PCA classification of the isolates from Simaisma mangroves, A) PCA, B) percentage of variance explained by PCA.

Fig. 7B shows the variation of the principal components for the mangroves samples, with PC1, PC2, and PC3 accounting for 28%, 15%, and 12% of the data variability respectively, resulting in a total of 55%. According to Fig. 7A, the PCA analysis of the mangroves samples resulted in the identification of four clusters. Cluster I is composed of the *V. alginolyticus* strains. Cluster II comprises the starins of *Sh. Putrefaciens*, while Cluster III is formed by strains of *Virgibacillus* spp.*, O. baumannii* and *Halomonas sp*. Cluster IV is composed of *Virgibacillus sp* strains. However, one of each of *P. agglomerans, V. dokdonensis*, and *H. Hydrothermalis* are not included in the groups as they are found in distance from other strains.

**Fig. 8:**
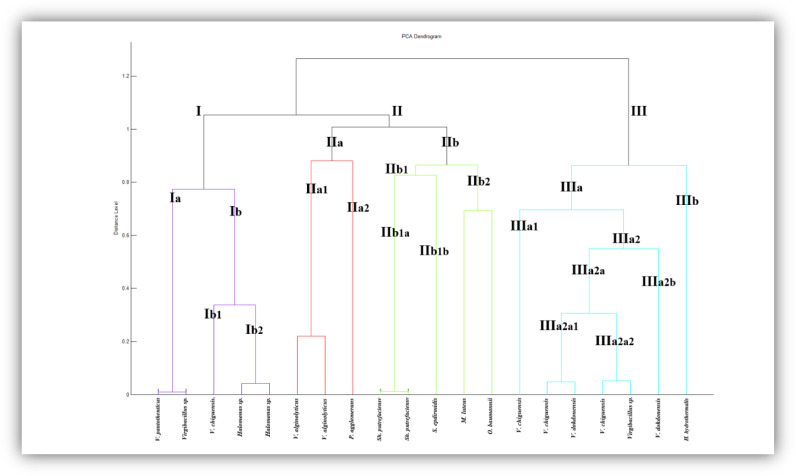
PCA dendrogram of all strains isolated from mangroves.

Fig. 8 shows the dendrogram of mangroves isolates, revealing that the three main clusters (I, II, and III) are further divided into multiple clades. Specifically, Cluster I is composed of five isolates, Cluster II contains eight isolates, and Cluster III is comprised of seven strains. Cluster I is divided into two clades Ia and Ib. Ia clade contains one strain of each *V. pantothenticus* and *Virgibacillus sp*. Ib clade is divided into Ib1 and Ib2 sub-clades. Ib1 holds *V. chiguensis* strain and Ib2 has two *Halamonas sp.* strains. Cluster II is composed of two clades namely, IIa and IIb. The clade IIa is further divided into two subclades (IIa1 and IIa2). The subclade IIa1 comprises two *V. alginolyticus* strains, while the subclade IIa2 is composed of one *P. agglomerans* strain. The clade IIb contains two subclades IIb1 and IIb2. The subclade IIb1 is composed of two *Sh. putrefaciens* strains and one strain of *S. Epdiermidis*. IIb2 covers two strains *M. luteus* and *O. baumannii*. Cluster III presents two clades IIIa and IIIb. IIIa clade shows two sub-clades IIIa1 and IIa2. IIIa1 has one strain of *V. chiguensis*, whereas IIIa2 is divided to two sub-clades IIIa2a and IIIa2b. IIIa2a divides more to IIIa2a1 containing one *V. chiguensis* and one *V. dokdonensis*, as well as IIIa2a2 which is composed of one *V. chiguensis* and one *Virgibacillus sp*. IIIa2b contains one *V. dokdonensis* strain. However, IIIb is formed of one *H. Hydrothermalis* strain.

### 3.4 Investigation on the potential of mineral formation by the isolated strains

MD1 medium employed in this work allows the enrichment of the successive cultures with bacteria, which are halophilic, heterotrophic and aerobic [10]. In this study, the potential of the isolated strains to form minerals was performed using MD1, MD1P and MD1Y. The Results of their potential to mediate mineral formation are shown in Table 2.

**Table 2:** Investigation of the potentials of mineral formation by the isolated bacterial strains: (-): no minerals are formed. Average number of crystals/mm^2^, +: 1–15, ++: 16–50.

No.	Code	ID	MD1	MD1Y	MD1P
1.	MC104	*Virgibacillus chiguensis*	**–**	**–**	**+**
2.	MC113	*Shewanella putrefaciens*	**–**	**–**	**++**
3.	MC1F32	*Virgibacillus sp.*	**–**	**–**	**+**
4.	MC1F33	*Virgibacillus pantothenticus*	**–**	**+**	**++**
5.	MC211	*Virgibacillus dokdonensis*	**–**	**+**	**++**
6.	MC223	*Virgibacillus chiguensis*	**–**	**+**	**++**
7.	MC224	*Halomonas sp.*	**–**	**–**	**–**
8.	MC2251	*Halomonas sp.*	**–**	**–**	**–**
9.	MC23	*Halomonas Hydrothermalis*	**–**	**–**	**–**
10.	MC231	*Staphylococcus epidermidis*	**–**	**–**	**+**
11.	MC233	*Virgibacillus dokdonensis*	**–**	**+**	**++**
12.	MC234	*Virgibacillus sp.*	**–**	**+**	**++**
13.	MC2F021	*Pantoea agglomerans*	**–**	**–**	**–**
14.	MC2F022	*Oceanimonas baumannii*	**–**	**–**	**–**
15.	MC2F111	*Vibrio alginolyticus*	**–**	**–**	**++**
16.	MC2F13	*Vibrio alginolyticus*	**–**	**+**	**++**
17.	MC2F211	*Virgibacillus dokdonensis*	**–**	**+**	**++**
18.	MC2F212	*Virgibacillus chiguensis*	**–**	**–**	**+**
19.	MC2F31	*Shewanella putrefaciens*	**–**	**+**	**++**
20.	MC2F34	*Micrococcus luteus*	**–**	**–**	**+**
21.	DFC02	*Virgibacillus chiguensis*	–	–	+
22.	DFC03	*Virgibacillus marismortui*	+	+	++
23.	DFC04	*Virgibacillus marismortui*	–	+	++
24.	DFC22	*Bacillus licheniformis*	–	–	–
25.	DFD111	*Bacillus subtilis*	–	–	–
26.	DFD12	*Bacillus lichenformis*	–	–	–
27.	DFD121	*Bacillus subtilis*	–	–	–
28.	DFD32	*Bacillus swezeyi*	–	–	–
29.	DFL02	*Virgibacillus chiguensis*	–	–	+
30.	DFL03	*Bacillus sp.*	–	–	–
31.	DFL04	*Bacillus seohaeanensis*	–	–	–
32.	DFL32	*Bacillus lichenformis*	–	–	–
33.	DFM01	*Vibrio alginolyticus*	–	–	+
34.	DFM03	*Vibrio alginolyticus*	–	–	+
35.	KA102	*Vibrio alginolyticus*	–	–	+
36.	KA103	*Salinivibrio proteolyticus*	–	+	+
37.	KA104	*Salinivibrio proteolyticus*	–	–	+
38.	KA105	*Salinivibrio proteolyticus*	–	–	+
39.	KA106	*Salinivibrio proteolyticus*	–	–	+

Based on the data presented in Table 2, it can be inferred that the use of MD1P medium resulted in the highest mineral formation capability for the studied strain compared to other tested media. Therefore, MD1P can be considered as the most recommended medium for investigating the mineral-forming ability of the strain initially isolated from Sabkhas and mangrove sediments in Qatar, which is a great novelty in this field.

Most of the mineral forming bacterial strains belong to the genera *Salinivibrio, Virgibacillus* and *Vibrio,* confirming earlier findings [7,31,36,37]. Remarkably, two strains of *Shewanella putrefaciens* (MC113 and MC2F31) obtained from Smeisma mangroves have been found to possess the ability to facilitate the formation of carbonate minerals. This finding adds to the previous reports, including the study by Chubar et al. (2015) [38], which demonstrated that *Shewanella putrefaciens* can mediate the formation of both manganese phosphate and manganese carbonate. Bacteria possess diverse mechanisms to actively influence and induce mineral formation [39]. One such mechanism involves the production of extracellular polymeric substances (EPS), which act as a matrix for mineral nucleation and growth [40]. Within the EPS, the presence of the carbonic anhydrase enzyme facilitates the localized conversion of carbon dioxide to bicarbonate ions [31]. This enzymatic activity increases the availability of bicarbonate ions, promoting mineral precipitation and contributing to the controlled formation of carbonate minerals [41].

**Fig. 9:**
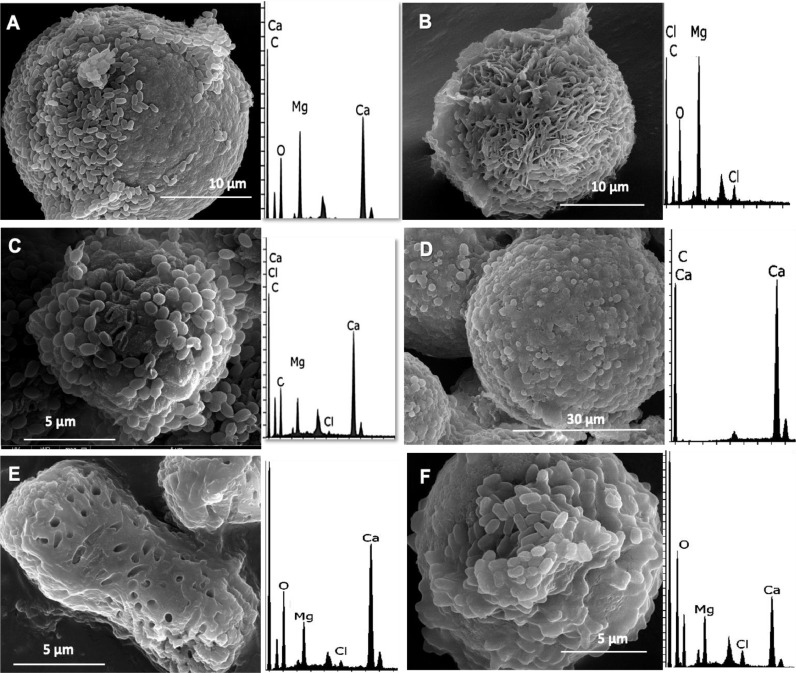
Representative SEM images and EDX spectra of the recovered minerals, A**)** Mg-calcite crystal recovered from pure cultures of *Virgibacillus marismortui* (DFC03), B) Calcium carbonate crystal recovered from pure cultures of *Vibrio alginolyticus* (MC2F13), C) Hydromagnesite crystal recovered from pure cultures of *Virgibacillus dokdonensis* (MC211), D) Calcium carbonate crystal recovered from pure cultures of *Salinivibrio proteolyticus* (KA104), E) Mg-calcite crystal recovered from pure cultures *Virgibacillus sp.* (MC234) and F) Mg-Calcite crystal recovered from pure cultures of *Shewanella putrefaciens* (MC2F31).

The SEM/EDX analysis of the minerals recovered from the pure cultures allowed the visualization of the formed mineral, as clear evidence and rough estimation of their elemental composition. The results of the analysis showed the formation of various types of carbonate minerals in the pure bacterial cultures, including calcium carbonates, hydromagnesite, and magnesium calcites. These minerals were found to have variable incorporation of magnesium into their crystal structure, as demonstrated in Fig. 9.

The potential of the corresponding strains to incorporate magnesium is then clearly evidenced. However, the variability in the incorporation of magnesium into the carbonate crystals observed in the SEM/EDX analysis suggests that the isolates may exhibit different mechanisms for mineral formation. Some of them, may lead to formation of precursors of dolomite at the ambient conditions. This variability could be explained by variability in the metabolic pathways or enzymes involved in carbonate precipitation, as well as differences in the environmental conditions under which the isolates were cultured. Further studies could be conducted to investigate these mechanisms and their implications for biomineralization [42].

## 4 Conclusion

Demonstrating the diversity of the mineral-forming bacteria is actually a necessity in biogeochemistry studies in sabkhas and sediments because these bacteria play a key role in the cycling of minerals and nutrients in these environments. In this study, a collection of aerobic bacterial strains isolated form decaying mats and living mats of Qatari sabkhas as well as from one Qatari mangroves site demonstrated a high diversity in their protein profiles, even within the same genus or species. Their close or far similarities should be attributed to their adaptation developed with time at specific environmental conditions. In pure cultures, the composition of the medium was found to be essential for formation of the minerals. Hence the composition of growth media should be considered for investigation the potential of mineral forming bacteria. The present work evidenced the biodiversity and the relationships with the adaptation of mineral-forming bacteria in the microenvironment of the cells. The use of MALDI-TOF MS approach was shown efficient to demonstrate the diversity of this type of bacteria and the diversity of their adaptation in the harsh conditions of Qatar environments, showing that the continuous adaptation dynamics would ensure its role played in Qatari extreme environments in capturing CO_2_.

## Funding

Open Access funding provided by the Qatar National Library.

## CRediT authorship contribution statement

**Toka Mahmoud Farhat:** Investigation, Data curation, Writing – original draft. **Zulfa Ali Al Disi:** Conceptualization, Visualization, Investigation, Data curation, Formal analysis, Writing – original draft. **Mohammad Yousaf Ashfaq:** Investigation, Visualization, Formal analysis. **Nabil Zouari:** Conceptualization, Methodology, Validation, Supervision, Writing – review & editing.

## Declaration of Competing Interest

The authors declare that they have no known competing financial interests or personal relationships that could have appeared to influence the work reported in this paper.

## Data Availability

No data was used for the research described in the article.
